# Genomic Prediction of Grain Yield and Drought-Adaptation Capacity in Sorghum Is Enhanced by Multi-Trait Analysis

**DOI:** 10.3389/fpls.2019.00997

**Published:** 2019-07-31

**Authors:** Julio G. Velazco, David R. Jordan, Emma S. Mace, Colleen H. Hunt, Marcos Malosetti, Fred A. van Eeuwijk

**Affiliations:** ^1^Department of Plant Breeding, EEA Pergamino, National Institute of Agricultural Technology (INTA), Pergamino, Argentina; ^2^Biometris – Mathematical and Statistical Methods, Wageningen University and Research, Wageningen, Netherlands; ^3^Queensland Alliance for Agriculture and Food Innovation, Hermitage Research Facility, The University of Queensland, Warwick, QLD, Australia; ^4^Department of Agriculture and Fisheries, Hermitage Research Facility, Queensland Government, Warwick, QLD, Australia

**Keywords:** genomic prediction, multi-trait analysis, sorghum, auxiliary trait, grain yield, stay-green, blended kinship matrix, BLUP

## Abstract

Grain yield and stay-green drought adaptation trait are important targets of selection in grain sorghum breeding for broad adaptation to a range of environments. Genomic prediction for these traits may be enhanced by joint multi-trait analysis. The objectives of this study were to assess the capacity of multi-trait models to improve genomic prediction of parental breeding values for grain yield and stay-green in sorghum by using information from correlated auxiliary traits, and to determine the combinations of traits that optimize predictive results in specific scenarios. The dataset included phenotypic performance of 2645 testcross hybrids across 26 environments as well as genomic and pedigree information on their female parental lines. The traits considered were grain yield (GY), stay-green (SG), plant height (PH), and flowering time (FT). We evaluated the improvement in predictive performance of multi-trait G-BLUP models relative to single-trait G-BLUP. The use of a blended kinship matrix exploiting pedigree and genomic information was also explored to optimize multi-trait predictions. Predictive ability for GY increased up to 16% when PH information on the training population was exploited through multi-trait genomic analysis. For SG prediction, full advantage from multi-trait G-BLUP was obtained only when GY information was also available on the predicted lines *per se*, with predictive ability improvements of up to 19%. Predictive ability, unbiasedness and accuracy of predictions from conventional multi-trait G-BLUP were further optimized by using a combined pedigree-genomic relationship matrix. Results of this study suggest that multi-trait genomic evaluation combining routinely measured traits may be used to improve prediction of crop productivity and drought adaptability in grain sorghum.

## Introduction

Water scarcity in rain-fed cropping systems is a major challenge to a world of increasing food demand (UNCTAD, 2011). In this context, grain sorghum (*Sorghum bicolor* L. Moench) is a cereal crop that can play an important role for sustainable farming, as it is particularly resilient to stress conditions caused by drought and erratic rainfall (Paterson et al., 2009). This crop is a staple food in semi-arid regions of the world and is used for feed globally (Acquaah, 2012). Grain yield is the primary trait in sorghum, as it is a key measure of crop productivity and profitability of farmers. Another important trait is stay-green; a complex drought-adaptation mechanism associated with increased yield in environments where post-flowering drought occurs frequently (Borrell et al., 2000; Jordan et al., 2003). Accordingly, most efforts for increasing genetic progress in grain sorghum should concern both characteristics (Jordan et al., 2012; Borrell et al., 2014). Further improvement in productivity and drought adaptability requires knowledge-based selection strategies that efficiently exploit available phenotypic and genotypic information in sorghum breeding programs.

Selection of complex quantitative traits can be based on statistical methods that combine phenotypes and high-density marker data to predict genetic merit. This form of marker-assisted selection, known as genomic selection (Meuwissen et al., 2001), has been successfully implemented in animal and plant breeding (Meuwissen et al., 2016; Crossa et al., 2017). Several genomic prediction methods have been developed, including Bayesian regression (Gianola et al., 2009) and semiparametric approaches (de los Campos et al., 2010). An alternative method for genomic prediction within the linear mixed model framework is termed genomic best linear unbiased prediction or G-BLUP (VanRaden, 2008). This method is usually preferred in practice because it is simple to implement and computationally less demanding than competing procedures (Gianola et al., 2014). Moreover, G-BLUP is expected to perform similar to other models for prediction of complex agronomic traits such as grain yield (Heslot et al., 2012; Wimmer et al., 2013), which are typically affected by a large number of small-effect genes (Schön et al., 2004). Independent of the model used, one of the main features of genomic selection is that genetic merit can be predicted for selection candidates that have not yet been phenotyped. This application is particularly promising for reducing evaluation cost and generation interval in the sorghum breeding pipeline, where parental lines of commercial hybrids are currently selected on the base of extensive field progeny testing. Moreover, development of female lines as well as hybrid seed production is based on the use of cytoplasmic-genetic male sterility, which requires extra time and human resources. Despite the potential of genomic selection to increase rates of genetic gain in sorghum, studies on the application of genomic models are limited compared to other cereal crops, such as maize, wheat, and rice (Kulwal, 2016). A first genomic selection study in grain sorghum was reported by Hunt et al. (2018) for prediction of test-cross performance in individual trials. Velazco et al. (2019) investigated different genomic models including pedigree information for across-environment prediction of parental breeding values in productivity and adaptability traits.

Most of genomic selection studies, including the ones mentioned above, have been based on separate analysis of individual traits. However, selection decisions in plant breeding programs typically rely on several measured characters. The joint analysis of multiple traits (MT) can increase the accuracy of genetic evaluations relative to single-trait (ST) analysis by exploiting information from correlated characters (Henderson and Quaas, 1976). The potential gain in accuracy depends on the strength of genetic and environmental correlations between traits. The benefit is expected to increase for lowly heritable traits, when analyzed together with strongly correlated traits of higher heritability (Thompson and Meyer, 1986). Additionally, MT models are able to reduce selection bias or culling bias introduced by contemporary or sequential selection on correlated traits, which are ignored by ST analysis (Mrode, 2005). The extension of MT analysis into the context of genomic prediction methods has been studied using real and simulated data (e.g., Calus and Veerkamp, 2011; Jia and Jannink, 2012). MT genomic models can be used to combine information from correlated traits and from relatives in an efficient way. When the breeder is interested in response to selection of a single target trait, other secondary or auxiliary traits can be incorporated in the prediction model to provide additional information on the primary character. Consequently, more phenotypes recorded on the reference population can be potentially exploited to assist predictions of the target trait in the testing population.

Genome-based MT analysis has been applied for breeding in hybrid crops of other major cereals like maize, rice and wheat (e.g., dos Santos et al., 2016; Wang et al., 2017; Schulthess et al., 2018). In sorghum, MT genomic prediction has been implemented only in biomass-type genotypes using a pre-breeding population (Fernandes et al., 2018). Here, we present a first study on the potential of exploiting trait associations for genomic prediction in advanced breeding material of grain sorghum. Our research is developed in the context of prediction for broad adaptation using testcross performance data across dryland sorghum production environments in Australia.

The aims of this study were to investigate if multi-trait analysis improves across-environment genomic predictions for grain yield and stay-green in sorghum, and to identify the combinations of traits that optimize results in different prediction scenarios. To attain these objectives, the optimal combination of traits was empirically determined for each scenario by evaluating the gain in prediction quality of alternative MT models relative to the ST model. In addition, we explored if the performance of best-predictive MT genomic models can be further enhanced by incorporating pedigree information.

## Materials and Methods

### Data

The dataset used in this study is part of the sorghum breeding program of female parental lines conducted by the University of Queensland and the Department of Agriculture and Fisheries in Queensland, Australia. The phenotypic records consisted of 26 testcross performance trials where a total of 646 female lines were tested in hybrid combinations across 12 locations between 2008 and 2014. Phenotypes of 2645 testcross hybrids were used to assess female lines performance across a target population of environments (TPE) covering the main sorghum cropping region of Australia. The series of trials belongs to an advanced stage of yield testing (AYT), where measurements are taken from relatively large plots. More details on field layout and structure of the dataset are given in Velazco et al. (2019). Four productivity and adaptability traits routinely measured in advanced testing were considered for this study: grain yield (GY), stay-green (SG), plant height (PH), and flowering time (FT). GY is the main target trait with direct economic value driving selection. SG is an integrated drought-adaptation trait that is expressed as delayed leaf senescence, which is a consequence of improved water balance in the plant under post-flowering drought stress (Borrell et al., 2014). This functional SG phenotype is also considered an important trait since it is associated with enhanced crop productivity in water-limited seasons (Jordan et al., 2003, 2012). Given that SG expression depends on the occurrence of terminal drought conditions, records of this trait were available at nine trials and for 603 lines in the present dataset. While PH and FT are mainly selected in earlier breeding stages to reduce extreme variation, these traits are considered in advanced testing to ensure appropriate agronomic type for commercial production (Jordan et al., 2011).

All the female lines were genotyped using an integrated DArT and genotyping-by-sequencing (GBS) methodology involving complexity reduction of the genomic DNA to remove repetitive sequences using methylation sensitive restriction enzymes prior to sequencing on Next Generation sequencing platforms (DArT)^1^. The sequence data generated were then aligned to the most recent version (v3.1.1) of the sorghum reference genome sequence (Paterson et al., 2009; McCormick et al., 2018) to identify SNP (Single Nucleotide Polymorphism) markers. SNPs with minor allele frequency lower than 2.5% or more than 20% of missing values were discarded. Missing genotypes were imputed based on random sampling from marginal allele distributions using the synbreed package (Wimmer et al., 2012) in R (R Core Team, 2018). After quality filtering, 4781 evenly spaced SNPs remained for analysis.

Inbred parent lines were derived from pedigree breeding methods resulting in a highly structured breeding population. Pedigree data was available for the female lines and 499 ancestors tracing back 28 generations.

### Single-Trait Analysis

Univariate analysis of each trait was performed within the REML-based mixed model framework using a stage-wise approach. In the first stage, adjusted testcross means were computed per trial after correcting for design factors and spatial field variation. For this, we used a novel flexible method for spatial analysis of individual trials based on P-splines (see Velazco et al., 2017; Rodríguez-Álvarez et al., 2018b; for details). The specific model applied in the first stage is described in Velazco et al. (2019).

In the second stage, spatially adjusted testcross means from each trial were jointly analyzed to estimate line means across testers and environments. The model is as follows:

(1) $$y_{ijk}\text{ = μ + }{\text{L}}_{i}\text{ + }{\text{M}}_{j}\text{ + }{\text{E}}_{k}\text{ + }{\text{LM}}_{ij}\text{ + }{\text{LE}}_{ik}\text{ + }{\text{ME}}_{jk}\text{ + }{\text{LME}}_{ijk},$$

where *y*_*ijk*_ represents the best linear unbiased estimation (BLUE) of the *i*-th female line crossed with the *j*-th male tester in the *k*-th environment, which was fitted by a random line effect (L*_*i*_*), a fixed male tester effect (M*_*j*_*), a fixed environmental effect (E*_*k*_*), and all possible interactions between these factors. Since line effects were considered random, all the interactions involving L*_*i*_* were random, while ME*_*jk*_* was fixed. Note that LME*_*ijk*_* includes the error of genotype mean. All random effects were assumed independent homoscedastic and normally distributed with zero mean.

Due to shrinkage properties of BLUP (Robinson, 1991), random line effects are contracted toward the mean of the line population. Given that not all lines were crossed with the same number of testers or evaluated in the same number of trials, the amount of shrinkage is different for each BLUP of L*_*i*_*. Moreover, using BLUP(L*_*i*_*) as response variable in the genomic prediction model is problematic because it would result in double shrinkage of predicted breeding values. Therefore, to eliminate shrinkage in line BLUPs before the genomic prediction stage, we applied the deregression procedure of Garrick et al. (2009). This correction relies on individual reliabilities of BLUP(L*_*i*_*), as obtained by inverting the coefficient matrix of the mixed model equation (Meyer, 1989).

The following G-BLUP model was used to predict parental breeding values of female lines from progeny performance:

(2) y  =  1μ  + Zg  +  e,

where the vector **y** contains deregressed BLUPs of L*_*i*_* derived from the second stage; **1** is a vector of ones with associated general mean μ; **Z** is a design matrix allocating deregressed BLUP(L*_*i*_*) to genomic effects; **g** is the vector of additive genomic effects with distribution **g** ∼ *N*(0, **G**$\sigma_{𝐠}^{2}$), where $\sigma_{𝐠}^{2}$ is the additive genomic variance and **G** is the genomic relationship matrix as computed with the first method of VanRaden (2008); and **e** is the vector of residuals assuming **e** ∼ *N*(0, **D**$\sigma_{𝐞}^{2}$), where $\sigma_{𝐞}^{2}$ is the residual variance and **D** is a diagonal weighting matrix accounting for heterogeneous residual variances due to differences in individual reliabilities of deregressed BLUP(L*_*i*_*) (VanRaden, 2008; Garrick et al., 2009).

Given the variance components from the ST prediction model, the narrow-sense heritabilities (*h*^2^) of line means were obtained as: $h^{2}=\sigma_{g}^{2}/\left(\sigma_{g}^{2}+\sigma_{e}^{2}\right)$. Note that $\sigma_{e}^{2}$ comprises non-additive genetic effects and true errors associated with line mean estimates.

### Multi-Trait Analysis

For joint MT analysis models (1) and (2) were extended using multivariate mixed models. We present a general formulation for any combination among traits T = (GY, SG, PH, FT). The multi-trait case of model (1) can be represented in vector notation as:

(3) $$y_{ijk}\text{ = μ + }L_{i}\text{ + }M_{j}\text{ + }E_{k}\text{ + }LM_{ij}\text{ + }LE_{ik}\text{ + }ME_{jk}\text{ + }LME_{ijk},$$

where in this case **y***_*ijk*_* is a vector collecting spatially adjusted BLUEs of multiple traits T from separate univariate analyses in stage one; and **L***_*i*_*, **LM***_*ij*_*, **LE***_*ik*_*, **LME***_*ijk*_* are the vectors of multi-trait random effects with respective assumed distributions **L***_*i*_* ∼ MVN(**0**, **I**_*L*_ ⊗ **Σ**_*L*_), **LM***_*ij*_* ∼ MVN(**0**, **I**_*LM*_ ⊗ **Σ**_*LM*_), **LE***_*ik*_* ∼ MVN(**0**, **I**_*LE*_ ⊗ **Σ**_*LE*_), **LME***_*ijk*_* ∼ MVN(**0**, **I**_*LME*_ ⊗ **Σ**_*LME*_), where **Σ***_*q*_*, for *q* = L, LM, LE, LME, is a covariance matrix among traits and ⊗ is the Kronecker product operator. For all random effects, the matrix **Σ***_*q*_* was modeled as unstructured, allowing for unequal variances across traits and specific covariances for each pair of traits.

The multi-trait G-BLUP model was defined as:

$$y_{T}\text{ = }1_{T}{\text{μ}}_{T}\text{ + }Z_{T}g_{T}\text{ + }e_{T},$$

where the subscript refers to traits T; **y**_*T*_ is now a multi-trait vector of deregressed BLUP(L*_*i*_*), ordered as lines within traits, obtained from joint multivariate analysis using model (3); **g**_*T*_ is the vector of multi-trait additive genomic effects with distribution **g**_*T*_ ∼ MVN(**0**, **G**⊗ **Σ***_*g*_*); and the multivariate residual effects were assumed **e**_*T*_ ∼ MVN[**0**, (**I***_*e*_* ⊗ **Σ***_*e*_*)**D**_*T*_], where matrix **D**_*T*_ has diagonal elements containing weights based on individual reliabilities of deregressed BLUP(L*_*i*_*) for each trait, as obtained from the second stage of multi-trait analysis (model 3). The covariance matrices among traits for additive genomic effects (**Σ***_*g*_*) and residuals effects (**Σ***_*e*_*) were assumed unstructured.

We also considered multi-trait prediction models fitting a kinship matrix that combines pedigree and genomic information. For this, we extended the BLUP method advocated by Velazco et al. (2019) to the multi-trait context. The method, referred as K-BLUP, uses a combined kinship matrix formed as **K** = *w***A** + (1 − *w*)**G_*s*_**, where **A** is the numerator relationship matrix among lines computed from the full pedigree data and **G_*s*_** is a scaled matrix **G** that is compatible with **A** in reference to the base breeding population (Vitezica et al., 2011; Christensen, 2012). The weighting term *w* can be interpreted as the fraction of additive genetic variance that is not captured by SNPs and is explained by familial relationships. In this case, the vector **g**_*T*_ has distribution MVN(**0**, **K**⊗ **Σ***_*g*_*), and collects additive genomic effects as well as residual polygenic effects. Under our approach, the optimal *w* is empirically determined in order to maximize predictive ability after evaluating a sequence of candidate weights within the range 0 < *w* < 1. To assess the benefits of including additional pedigree information, the best-predictive multi-trait model using conventional G-BLUP (i.e., setting *w* = 0) was compared to the equivalent optimized multi-trait K-BLUP model.

### Model Fitting

Spatial analyses in the first stage were performed with the R package SpATS (Rodríguez-Álvarez et al., 2018a), which is publicly available from CRAN^2^. Uni- and multivariate models in the second and the genomic prediction stages were fitted by REML using the average information algorithm as implemented in ASReml-R (Butler et al., 2017). We used the correlation form parameterization of the unstructured covariance matrix available in ASReml-R to obtain direct estimates of trait correlations and corresponding standard errors.

### Prediction Scenarios

**FIGURE 1:**
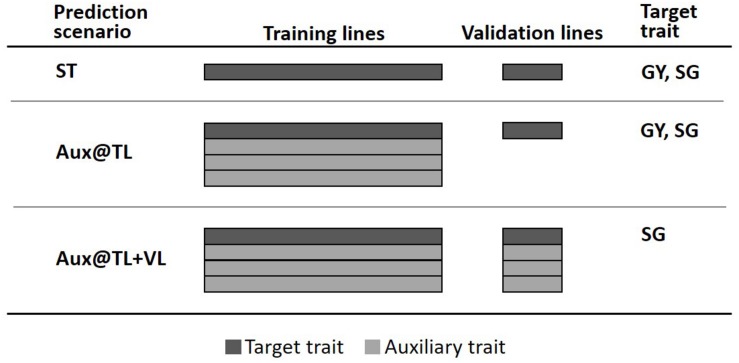
Fivefold cross-validation schemes representing three prediction scenarios: ST, where only data of the target trait in the training lines set (TLS) are used for prediction in the validation lines set (VLS); Aux@TL, where data of auxiliary traits in TLS are included for prediction of the target trait in VLS; and Aux@TL + VL, where data of auxiliary traits in both TLS and VLS are included for prediction of the target trait in VLS.

To assess the value of multi-trait genomic analysis in sorghum, we considered the general predictive strategy where a single trait is the primary target of prediction (selection) and other auxiliary characters are used to potentially improve predictions of the target trait. Predictive performance of MT models was evaluated through fivefold cross-validation, where 80% of lines were randomly assigned to the training lines set (TLS) and the remaining 20% formed the validation lines set (VLS). We explored two potential scenarios for MT prediction (Figure 1). One scenario assuming that (new) lines in the VLS have not been evaluated in the field for any trait; therefore, phenotypes of auxiliary traits (and the target trait) were available only for the TLS (Aux@TL). A second MT prediction scenario assumes that lines of the VLS have been evaluated in the field, but for traits other than the target; therefore, phenotypes of auxiliary traits were available for both TLS and VLS (Aux@TL + VL). For comparison, we also considered ST prediction, where only records of the target trait in VLS are used for prediction.

The first MT prediction scenario was studied for two cases: when GY or SG were the traits of interest. However, the Aux@TL + VL scenario was considered only for SG as target trait. Given that one of the main purposes of the trial series used in this study is to obtain representative measurements of GY productivity, the fact that lines are field-tested for other traits, but not for GY, would imply atypical circumstances seriously affecting GY during an experiment. Moreover, extremely reduced or aborted grain production affects normal canopy development; hence, data of traits such as PH or SG should be discarded or considered with extreme caution. For these reasons, the Aux@TL + VL scenario was not considered when GY was the target of prediction, as it would represent an unrealistic or exceptional situation in the context of an advanced stage of sorghum testing.

### Validation of Genomic Prediction

In each prediction scenario, the performances of all possible MT models combining two, three, and the four traits were compared to that of the ST model, with the latter taken as benchmark. The response variables used for validation of genomic predictions were consistent with the models applied for analysis: **y**_*T*_ and **y** were used to validate $\tilde{\text{𝐠}}{}_{T}$ and $\tilde{\text{𝐠}}$, respectively. Model validation was evaluated by considering the predictive ability, unbiasedness, and accuracy of predictions in the VLS. Predictive ability was measured as the Pearson’s correlation (*r*_*PA*_) between $\tilde{g}{}_{T}$ ($\tilde{g}$) and **y**_*T*_ (**y**). Unbiasedness of genomic predictions was measured by the regression coefficient (*b*) of **y**_*T*_ (**y**) on $\tilde{g}{}_{T}$ ($\tilde{g}$), where *b* = 1 indicates an empirically unbiased predictor. Accuracy of predictions was assessed by computing the mean squared error of prediction (MSEP) from the linear regression. The evaluation of prediction models was based on average values over 20 replicates of each cross-validation scenario using the same random seed for all models.

## Results

The narrow-sense heritability estimates using ST G-BLUP are given in Table 1 along with the additive genetic and residual correlations between traits estimated by MT G-BLUP. Heritability estimates ranged from 0.36 to 0.76, with GY presenting the lowest *h*^2^. The heritability of SG was higher than that of GY, but lower than those of PH and FT. Additive genetic correlations were significant only for GY with SG and PH, while FT was statistically uncorrelated with the other traits. Significant residual correlations were estimated between GY and the rest of the traits as well as between PH and FT.

**TABLE 1:** Heritabilities (diagonal; in parentheses), additive genetic (above diagonal), and residual (below diagonal) correlations^a^ for grain yield (GY), stay-green (SG), plant height (PH), and flowering time (FT).

**Trait**	**GY**	**SG**	**PH**	**FT**
GY	(0.36)	**0.52**	**0.66**	−0.01
SG	**0.36**	(0.50)	0.03	−0.02
PH	**0.71**	0.04	(0.76)	−0.07
FT	−**0.48**	0.04	−**0.29**	(0.65)

*^*a*^Boldfaced correlations are significant based on approximate 95% confidence interval (Holland, 2006).*

**FIGURE 2:**
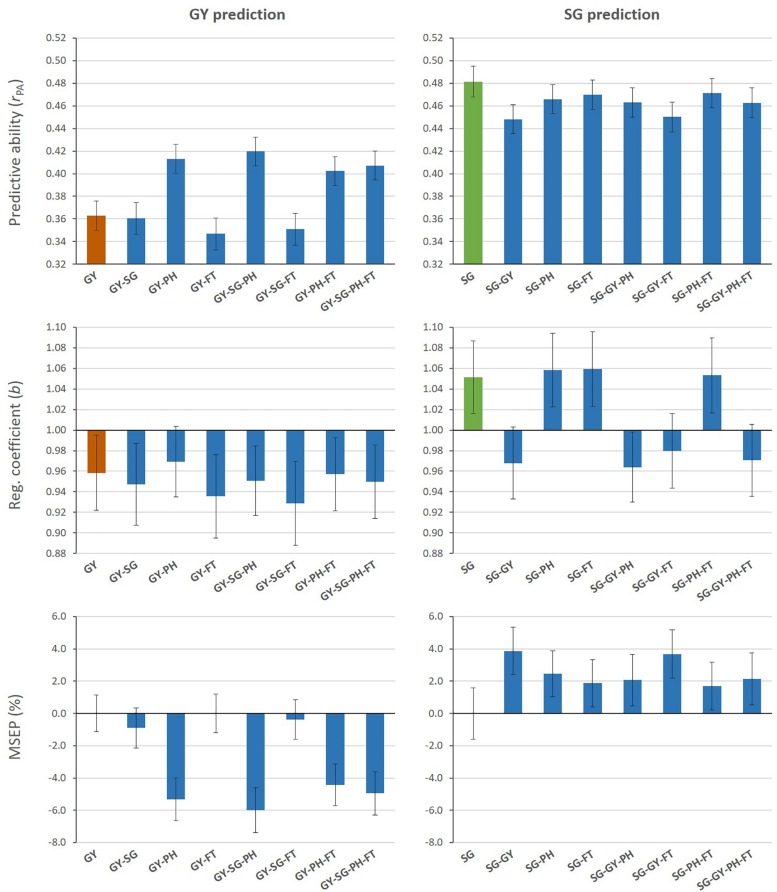
Mean values (and SD of 20 replicates) for predictive ability, regression coefficient, and relative MSEP from single- and multi-trait G-BLUP models using different combinations of grain yield (GY), stay-green (SG), plant height (PH), and flowering time (FT) data in the training lines set for prediction of GY (left) or SG (right).

Figure 2 shows measures of predictive performance from MT G-BLUP when additional information from auxiliary traits in the TLS was used to predict GY or SG (Aux@TL scenario). MT models that included PH data alone or combined with other auxiliary traits increased predictive ability for GY, compared with the ST model. The highest predictive ability represented a 16% increase and it was achieved by combining GY, SG and PH information. Regression coefficients (*b*) for all models were below 1 for GY, which indicates overestimation of genomic predictions. The average empirical bias of GY predictions from the ST model was slightly reduced only by the MT model including PH alone as auxiliary trait. The relative decrease (%) in MSEP by MT models was consistent with their increases in predictive ability; MT G-BLUP exploiting PH information had a greater impact on improving accuracy of genomic prediction for GY. When SG was the target of prediction, ST G-BLUP outperformed MT G-BLUP models in predictive ability. Even though prediction models incorporating GY data in the TLS reduced the absolute deviation of *b* from 1, these models tended to over-predict additive genetic values for SG (*b* < 1). The inclusion of auxiliary traits in TLS produced higher MSEP relative to using only SG data for prediction.

**FIGURE 3:**
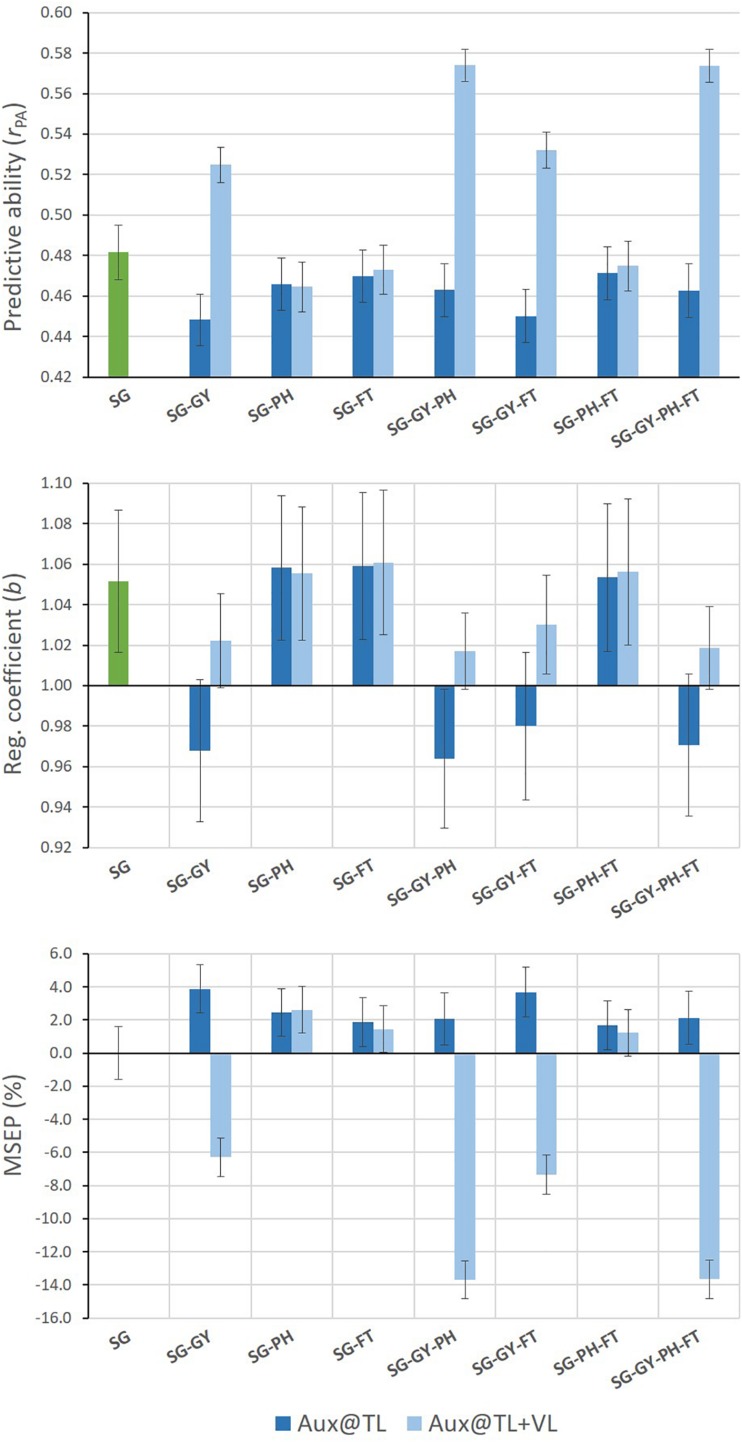
Mean values (and SD of 20 replicates) for predictive ability, regression coefficient, and relative MSEP for SG predictions from single- and multi-trait G-BLUP models using different combinations of grain yield (GY), stay-green (SG), plant height (PH), and flowering time (FT) data only in the training lines set (Aux@TL), and in both the training and validation lines sets (Aux@TL + VL).

Different results were found for SG predictions when MT G-BLUP models used auxiliary trait records on both TLS and VLS (Figure 3). Under this prediction scenario, all MT models including GY as auxiliary trait improved predictive abilities for SG. Relative increases of up to 19% in predictive ability were observed for MT models adding combined GY-PH or GY-PH-FT information from TLS and VLS. Moreover, predictions from these models tended to be less biased than those from ST G-BLUP and over-prediction was eliminated. Finally, MSEP for SG was consistently reduced when GY information on VLS was incorporated by MT G-BLUP models.

After evaluating predictive performance of G-BLUP method, we explored the use of a combined pedigree-genomic matrix **K** for optimization of multi-trait genomic prediction. Table 2 presents results from the best-predictive MT G-BLUP models and from the optimized MT K-BLUP models for the scenarios where MT analysis outperformed ST analysis (Aux@TL for GY and Aux@TL + VL for SG). In these prediction scenarios, the optimal use of combined trait information, from a predictive perspective, resulted from exploiting genetic correlations among GY, SG, and PH (Figures 2, 3). When GY was the target trait, validation results of MT G-BLUP were improved by MT K-BLUP (with **K** using *w* = 0.25) for all evaluation criteria to a marginal extent. Under the prediction scenario for SG, bias and MSEP from multi-trait models were slightly higher when **K** (using *w* = 0.30) was used instead of **G**. However, an additional 10% gain in predictive ability was obtained by MT K-BLUP in this scenario.

**TABLE 2:** Predictive abilities (*r*_*PA*_), regression coefficients (Bias), and relative MSEP from single-trait G-BLUP and K-BLUP, and from the best-predictive multi-trait G-BLUP and K-BLUP models for GY prediction using auxiliary traits data on training lines (Aux@TL) and for SG prediction using auxiliary traits data on both training and validation lines (Aux@TL + VL).

**Prediction scenario**	**Quality criterion**	**Single-trait**		**Multi-trait^a^**	
			
		**G-BLUP**	**K-BLUP**	**G-BLUP**	**K-BLUP^b^**
GY: (Aux@TL)	*r*_*PA*_	0.363 (0.013)	0.373 (0.014)	0.420 (0.013)	**0.429** (0.015)
	Bias (*b*)	0.958 (0.037)	0.965 (0.037)	0.951 (0.034)	**0.967** (0.039)
	MSEP (%)	0 (1.1)	−0.7 (1.1)	−6.0 (1.3)	−**6.6** (1.6)
SG: (Aux@TL + VL)	*r*_*PA*_	0.482 (0.013)	0.508 (0.015)	0.574 (0.008)	**0.630** (0.009)
	Bias (*b*)	1.052 (0.035)	1.074 (0.036)	**1.017** (0.019)	1.038 (0.022)
	MSEP (%)	0 (1.6)	2.7 (1.6)	−**13.7** (1.2)	−12.0 (1.2)

*The best value for each evaluation criterion is boldfaced. ^*a*^Multi-trait models combining GY, SG, and PH information, ^*b*^Using **K** = *w***A** + (1 − *w*)**G_*s*_** with optimal weights *w* = 0.25 and *w* = 0.30 for GY and SG prediction, respectively.*

## Discussion

This study intended to establish the value of using multi-trait analysis to improve across-environment genomic predictions for grain yield and stay-green in sorghum by exploiting information from auxiliary traits. Our approach to predict is consistent with selection for broad adaptation, where the set of trials is considered to be representative of the TPE. To determine the efficiency of this predictive method, multi-trait BLUP models were evaluated in terms of prediction quality measures for two cases: when the target trait and auxiliary traits were recorded only on relatives of the predicted lines; and when records of auxiliary traits were also available for the predicted lines *per se*. Multi-trait BLUP can be seen as a generalized (linear) selection index, with the additional abilities to account for unbalanced information from any set of relatives while properly adjusting for fixed effects in the data (Lynch and Walsh, 1998; Mrode, 2005). In our index, and from mixed model theory, data of several traits were optimally weighted based on the multi-trait genetic and residual covariance matrices in order to maximize accuracy of predicted genetic merit by BLUP estimation. An important decision is which auxiliary traits to include in a multi-trait model. In this article, we examined all possible combinations of available traits, and the optimal multi-trait BLUP model for each prediction scenario was empirically determined on the base of predictive outcomes. The present study considered the general situation where the breeding objective is to improve a single character with economic value (GY or SG), while other traits would contribute to that genetic goal. This is equivalent to assigning a relative economic weight of 1 to the target trait and 0 weights to auxiliary traits in a selection index. It is worth mentioning that, in a real breeding program, SG is unlikely to be considered as an independent target of selection since its economic value is linked to its positive effect on GY and reduced lodging under stressing conditions. Our research could be easily extended to the case of simultaneous improvement for GY and SG by constructing an index with non-zero economic weights for both traits. This would give predictive results that are intermediate of those presented in our research, with variations depending on the relative economic weights assigned (not shown). Given that defining the appropriate relative economic value of GY and SG is beyond the scope of the present study, here we presented the evaluation of multi-trait prediction for a target trait at a time without losing generality of results.

### Multi-Trait Genomic Prediction for Grain Yield

Genetic correlations between traits estimated in this research are consistent with previous results showing strong associations of GY with SG, PH, and FT in sorghum hybrids (Jordan et al., 2003). In our case, additive parental effects of GY were only correlated with additive effect of SG and PH, whereas associations between GY and FT were basically explained by residual effects. In addition, the estimated heritability for GY in our study was lower than for the rest of the traits. Based on these findings, and according to multivariate BLUP and selection index theories, prediction of breeding values for GY should mainly benefit by utilizing additional information from SG and PH, while introducing an uncorrelated trait, such as FT, would reduce prediction efficiency due to incorporation of sampling error. These expectations were corroborated by our predictive results from cross-validation when records of auxiliary traits were available for the training lines (Figure 2). Multi-trait models combining GY-SG-PH information produced the highest improvements in predictive ability for GY, representing up to 18% increase relative to ST G-BLUP (Table 2). These results are somewhat more promising than those previously reported for GY prediction in wheat (Rutkoski et al., 2016; Sun et al., 2017), maize (Lyra et al., 2017), and rye (Schulthess et al., 2016), which found null increases in predictive ability under equivalent prediction scenarios. The differences in response from using MT analysis are highly dependent of the experimental data and the genetic target of prediction. In our research, the inclusion of PH information in multi-trait models appeared to be essential for improving predictive ability, unbiasedness and accuracy of GY predictions. This may be attributed to the fact that PH presented the strongest additive genetic correlation and the largest difference in heritability with GY. The key role of these two factors for benefiting from multi-trait prediction of low-heritability traits has been demonstrated by simulation studies in plant and animal breeding contexts (Jia and Jannink, 2012; Guo et al., 2014). In addition, the present study assumes that the genetic target of selection is across-environment breeding value, which depends on overall heritabilities and trait correlations across the TPE. It should be considered, however, that the levels of heritability and correlations are variable among experiments due to the influence genotype-by-environment interaction, inducing variability of predictive results in specific trials.

### Multi-Trait Genomic Prediction for Stay-Green

Our validation results were different for SG prediction when only MT information on the training set was used. As expected from considering heritabilities and additive genetic trait correlations, predictive performance of the ST model was generally better than those of MT models. This may be explained because SG was genetically correlated only with GY, but this trait had the lowest heritability (Table 1). Under these circumstances, information from PH and FT could not be borrowed to predict SG, while GY data would be genetically less informative than the target trait itself. On the other hand, predictive results favored multi-trait genomic models when GY information was available for both the training lines and the predicted lines *per se*, which was reflected by increases in predictive ability and reductions in bias and MSEP of SG predictions (Figure 3). The comparison of both prediction scenarios for SG allowed an empirical assessment of the value of two different sources of information from correlated traits. When auxiliary traits are only available for the training set, the estimates of covariance parameters among traits in **Σ***_*g*_*, which are then used for prediction, contain information from the training lines exclusively. Alternatively, if records of auxiliary traits are also available for the validation set, better parameter estimates in **Σ***_*g*_* can be obtained for prediction of the target trait. This is not only because more data is used for parameter estimation, but mainly because the extra information contained in estimated trait correlations is directly sourced from the predicted lines. Our results for SG suggest that, when this extra information is exploited, the strength of correlations among traits is more important than the relative levels of trait heritability. This would explain why SG predictions were improved by multi-trait G-BLUP using GY data, even when this trait had lower heritability than the target trait. Moreover, predictive performance of the MT model combining SG, GY, and PH indicates that, despite SG and PH were uncorrelated, additional information from PH could be transmitted via GY to further enhance predictions of SG (Figure 3). Similar benefits of exploiting information from secondary traits in the validation set have been reported in crops for other traits, but not for SG (Rutkoski et al., 2016; Fernandes et al., 2018; Lado et al., 2018). Even though the link between stay-green trait and yield stability has been demonstrated in sorghum, maize and wheat (Christopher et al., 2008; Jordan et al., 2012; Gregersen et al., 2013), the predictive use of SG-GY association in cereals had not been studied yet in the context of genomic selection.

### Combining Pedigree and Genomic Information for Multi-Trait Prediction

This study explored if additional genealogical information from pedigree could further improve multi-trait genomic prediction. Merging pedigree and marker-based relationships has been shown to be beneficial for ST prediction in animals (e.g., Rodríguez-Ramilo et al., 2014; Ilska et al., 2017) and more recently in plant breeding (Velazco et al., 2019). In the context of multi-trait analysis, models relying exclusively on SNP information may give distorted estimates of genetic variances and correlations between traits, mainly due to incomplete linkage disequilibrium (LD) of markers with causal loci (de los Campos et al., 2015; Gianola et al., 2015). Pedigree information can be used to account for residual polygenic effects not traced by SNPs, capturing LD patterns between loci that are due to common ancestral identity. Our study showed that better predictions of GY and SG were obtained by multi-trait models using a weighted combination of **A** and **G** instead of **G** alone (Table 2). Multi-trait K-BLUP produced the highest increase in predictive ability (of about 30%) for the SG prediction scenario, where trait correlations seemed to play a more important role in model performance. This result suggests that the **K** matrix optimized estimates of genetic correlation between traits, from a predictive perspective. The same approach has been applied to infer trait correlations from multi-trait prediction models in chicken (Momen et al., 2017).

Here, we used a different optimal weight *w* to construct **K** according to the trait of interest for prediction (*w* = 0.25 and *w* = 0.30 to predict GY and SG, respectively). This is in line with the idea that genetic (genomic) similarities between relatives are actually trait-specific (Fernando and Gianola, 2018), which contrasts with the assumption of a common relationship matrix used in standard multi-trait G-BLUP. A more elaborate multi-trait G-BLUP model using trait-dependent weights has been recently proposed by Karaman et al. (2018) as a computationally less demanding alternative to multi-trait Bayesian methods. In this BLUP model, weights used to compute **G** are derived from posterior trait-specific (co)variance estimates of SNP effects, which are obtained from a previous Bayesian analysis. The authors found, based on simulations, that the benefits of their weighting method were generally not significant for the low-heritability trait that more closely fitted an infinitesimal model (using 500 QTLs). Alternatively, our multi-trait K-BLUP uses a more straightforward approach that assumes a common weight across the genome, while still relaxing the assumptions of conventional multi-trait G-BLUP by allowing the similarity matrix to vary across traits in order to optimize prediction of the target. Given the complexity of traits and the highly structured breeding population used for this study, genomic predictions are likely to rely more on familial relationships and less on information from specific SNPs in LD with QTL (Habier et al., 2007). In this context, the BLUP-based multi-trait models applied here are expected to perform well compared to more refined alternatives (Jia and Jannink, 2012; Haile et al., 2018).

### Implications of Multi-Trait Genomic Prediction for Grain Sorghum Breeding

While genotyping costs are being constantly reduced, the efficient use of phenotypic data becomes more relevant for plant breeding programs, since field phenotyping is still costly and labor-intensive. This work has shown for the first time how phenotypes of several traits routinely measured in grain sorghum breeding can be efficiently utilized through multi-trait analysis to assist genome-based selection of a target trait. We demonstrated empirically that genomic prediction of parental breeding values for GY benefits mainly from using PH as auxiliary trait. This trait is particularly promising in practice, since PH phenotype can be potentially measured in all trials, as opposed to SG, which can only be phenotyped under specific environmental conditions. In addition, high throughput field phenotyping technologies are expected to increase accuracy of PH measurement in sorghum (Wang et al., 2018), which could favor GY selection indirectly through multi-trait prediction. The potential of exploiting genetic association between GY and PH predictively brings a new perspective regarding selection strategies in advanced sorghum testing. Our results imply that, despite strong selection for appropriate height is generally imposed in early stages of breeding, multi-trait evaluation could be implemented for increasing the capacity to use remaining genetic variation in PH when selecting for GY. That is, selection based on multi-trait models is able to directly use the variation in GY that is not associated with PH, and can simultaneously exploit GY variability indirectly through the available variation in PH (within the acceptable range).

Based on findings from this study, when SG is the target of prediction, most advantage from multi-trait genomic analysis can be obtained only if predicted lines have been phenotyped for GY. This may be beneficial when the interest is to predict the genetic aptitude for drought-adaptation in lines that have been field-tested for GY performance but could not yet experience post-flowering water stress. Such scenario is compatible with selection for broad adaptation across the TPE, which is generally the best approach to deal with the largely unpredictable occurrence of drought in the Australian sorghum region (Chapman et al., 2000). Expression of functional SG can also be a consequence of reduced sink demand relative to source, due to low grain production at plant level (Henzell and Gillieron, 1973; Duncan et al., 1981). For this reason, Borrell et al. (2014) pointed out that simultaneous selection for SG and GY should be applied in sorghum breeding programs to correct for functional SG that is actually driven by low sink demand. This correction can be automatically performed by joint analysis of both traits using multi-trait genomic models. For instance, in our case, selection of parental lines for broad adaptation would be based on a predicted genetic score that is optimally derived by combining three sources of information: direct information from own GY breeding value in well-watered environments, and indirect information from SG and GY breeding value of relatives in water-limited environments.

## Conclusion

This study demonstrates, based on extensive breeding data, that there is potential to improve genome-based predictions of grain yield and stay-green traits in grain sorghum by using multi-trait genomic analysis. Results suggest that better predictive abilities and accuracies for GY prediction are obtained when PH information on the training lines is included in multi-trait genomic models. When SG is the target, the quality of predictions is likely to improve only if GY performance data is available for both the training and the predicted lines *per se* since, in this case, direct information from SG-GY genetic correlations is exploited predictively through multi-trait analysis. This article also shows, for the first time in plant breeding, how a similarity matrix using trait-specific combinations of pedigree and marker-based relatedness can further enhance multi-trait genomic prediction. Collectively, our results imply useful properties of multi-trait BLUP to evaluate alternative prediction schemes for genetic improvement of crop productivity and drought adaptability in grain sorghum. Given that the traits considered in this study are commonly measured in cereal breeding programs, findings presented here can be also relevant for practical implementation in other major crops.

## Data Availability

The raw data supporting the conclusions of this manuscript will be made available by the authors, without undue reservation, to any qualified researcher.

## Author Contributions

JV, DJ, MM, and FvE designed the research. JV performed the statistical analyses and wrote the manuscript. DJ and FvE edited the manuscript. DJ coordinated the experiments and data collection. CH and EM processed and prepared the dataset. All authors read and approved the final version of the manuscript.

## Conflict of Interest Statement

The authors declare that the research was conducted in the absence of any commercial or financial relationships that could be construed as a potential conflict of interest.

## Footnotes

1 http://www.diversityarrays.com

2 https://cran.r-project.org/package=SpATS
